# Assessment of β-human-derived chorionic gonadotrophic hormone (βhCG) and pregnancy-associated plasma protein A (PAPP-A) levels as predictive factors of preeclampsia in the first trimester among Iranian women: a cohort study

**DOI:** 10.1186/s12884-019-2526-x

**Published:** 2019-12-04

**Authors:** Maryam Honarjoo, Shahnaz Kohan, Elahe Zarean, Mohammad Javad Tarrahi

**Affiliations:** 1 Student research committee, School of nursing and midwifery, Isfahan University of Medical Sciences, Isfahan, Iran; 2 Associated professor, Nursing and Midwifery Care Research Center, Faculty of Nursing and Midwifery, Isfahan University of Medical Sciences, Isfahan, Iran; 3 Department of Obstetrics and Gynecology, Fetal Medicine Unit, Isfahan University of Medical Sciences, Isfahan, Iran; 4 Associated professor, Department of Epidemiology and Biostatistics, School of health, Isfahan University of Medical Sciences, Isfahan, Iran

**Keywords:** NT, PAPP-A, βhCG, First trimester of pregnancy, PE

## Abstract

**Background:**

Preeclampsia (PE) is a leading cause of maternal and perinatal mortality. There are controversial findings regarding the prediction of PE through the assessment of the Pregnancy-Associated Plasma Protein A (PAPP-A) and β-Human-Derived Chorionic Gonadotrophic hormone (βhCG) levels in the first trimester of pregnancy. Therefore, this cohort study was conducted to evaluate of PAPP-A and βhCG levels as predictive factors for PE development in the first trimester among Iranian women.

**Methods:**

In this cohort study, a total of 4605 volunteer Primigravida and Multigravida women were selected by the census from 16 randomly selected Health Centers in Isfahan, Iran, from July 2016 to June 2018. Eligible pregnant women participated in the study had already undergone fetal anomalies screening tests between 11 + 0 and 13 + 6 weeks of pregnancy and their PAPP-A and βhCG biomarkers were adjusted to the Multiples of the Median (MOM). MOM PAPP-A <  0.4 and MOM βhCG > 3 were considered abnormal. The samples were followed up until delivery. The biomarkers’ levels were compared in the two groups of women with and without PE and Relative risk (RR) and odds ratio (OR) of PE calculated.

**Results:**

In the PE group, the mean MOM PAPP-A was significantly lower (1 vs. 1.09 with *P* = 0.006) and MOM βhCG was significantly higher (1.51 vs. 1.14 with *P* = 0.001) than the group without PE. RR and OR for PE in subjects with MOM PAPP-A <  0.4 were reported as follows: RR = 2.49, (*p* = 0.001) and OR = 2.09, (*p* = 0.001). RR and OR for PE in subjects with MOM βhCG > 3 were also reported as follows: RR = 4.02, (*p* = 0.001) and OR = 5.65, (*p* = 0.001). Adjusted OR for MOM PAPP-A <  0.4 and MOM βhCG > 3 was obtained as follows: OR = 2.09 (*P* = 0.001) and OR = 5.65 (*P* = 0.001), respectively.

**Conclusion:**

The results of the study showed that the high levels of βhCG would cause 5.65 times increase and the low levels of PAPP-A would cause 2.09 times increase in the chance of developing PE.

## Background

Preeclampsia (PE) is a pregnancy-specific disorder affecting 2–8% of all pregnancies [1]. PE is considered a leading cause of maternal and perinatal mortality [2]. It is a multi-system disorder and may cause serious complications such as eclampsia, H (hemolysis) EL (elevated liver enzymes) LP (low platelet count) syndrome, pulmonary edema, renal failure, Disseminated Intravascular Coagulopathy (DIC), hypertension-associated encephalopathy and cortical blindness in the mother. In addition, PE causes many fetal complications such as stillbirth, Intrauterine Growth Restriction (IUGR), neurological problems, bronchopulmonary dysplasia, etc. [3].

Recent evidence suggests that the short-term consequences of PE only represent the tip of the iceberg since women suffering from this disorder are apt to develop type 2 diabetes, cognitive impairment and calcifications in the coronary arteries in the next three decades of life [2].

Early identification of women exposed to PE is regarded as a key aim of prenatal care [4] and the early onset of management strategies such as providing appropriate prenatal care is essential to minimize the undesirable outcomes [1].

To date, several biochemical markers have been used to evaluate the predictive factors of PE before the appearance of clinical symptoms. Several studies showed the association between the reduction of PAPP-A and βhCG levels in the first trimester and the development of adverse pregnancy consequences such as PE; however, some findings are controversial [3, 5–8]. Considering the heterogeneous results obtained from various studies, the relevancy of placental biomarkers has not yet been determined, as well as the differences in race and also the limitations introduced in the previous studies. Therefore, further researches with larger samples are needed to support the hypothesis that the subsequent pregnancy complications could be detected in the first trimester by abnormal serum levels of these biomarkers [9]. Thus, this cohort study was aimed to evaluate of PAPP-A and βhCG levels as predictive factors for PE development in the first trimester among Iranian women.

## Methods

This cohort study was conducted from July 2016 to June 2018. Accordingly, 25,618 pregnant women referred to 52 Isfahan Health Centers to receive prenatal care. Among them, 4605 eligible pregnant women were selected by the census from 16 health centers which randomly selected.

Pregnant women had already undergone combined fetal anomaly screening tests based on Iran’s national protocol between 11 + 0 and 13+ 6 weeks of pregnancy. This protocol includes ultrasound for measuring the Nuchal Translucency (NT) combined with the PAPP-A and freeβhCG levels in the mother’s serum. The gestational age was determined based on the Crown-Rump Length (CRL) in the ultrasound which performed in the first trimester. Then, the serum levels of biomarkers were adjusted to MOM using the information on gestational age, ethnicity, Body Mass Index (BMI), diabetes, and smoking status based on the local referrals.

Volunteer Iranian pregnant women were included in the study accordingly: women with a singleton pregnancy, using no drugs, narcotics, cigarettes, and alcohol, having no fetal abnormalities, as well as lacking diseases affecting the process of pregnancy such as lupus. The structural defects such as abdominal wall defects, neural tube defects or fetal abnormality were detected by an ultrasound anomaly scan, in the second-trimester screening, Moreover, women having home delivery, a neonate born with Down syndrome or visible anomaly were excluded from the study.

Preeclampsia was defined according to the guidelines of the American College of Obstetricians and Gynecologists, based on systolic blood pressure ≥ 140 mmHg or diastolic blood pressure ≥ 90 mmHg on 2 recordings at least 4 h apart, with the presence of proteinuria 300 mg in 24)h(or ≥ 1 protein on dipstick analysis, after 20 weeks of gestation in a woman with previously normal blood pressure [9].

The Obstetric and demographic characteristics of pregnant women were collected and recorded in the information forms at the beginning of the study. Also they were evaluated by the prenatalogist for confirming PE development. The Participants were followed up three times between 24 and 28 and 32–34 weeks of the pregnancy and immediately after childbirth.

Data was collected by completing the checklist based on the clinical examinations, birth records, prenatal ultrasounds, documents from computerized documentation systems and pregnant women with obtaining their consents. In order to evaluate the PAPP-A and βhCG levels in the first trimester to predict the PE, the mean of MOM PAPP-A and MOM βhCG was compared in the groups of women with PE and without PE. The values of MOM PAPP-A <  0.4 and MOM βhCG > 3 were considered abnormal [10, 11]. A comparison was made between the two groups of women with PE and without PE based on relative risk and PE odds ratios.

All statistical analysis was performed using SPSS software (version 16, SPSS, Inc., Chicago, IL, USA). Independent Samples t-test and Chi-Square test were used to determine the relationship between the levels of βhCG, PAPP-A biomarkers and PE. In order to calculate the relative risk and adjusted OR, the abnormal PAPP-A, and βhCG biomarkers and PE predictors, the Logistic Regression was used to adjust the results related to confounding variables of women’s age, spouse’s age, BMI, abortion history, The Fetal Growth Restriction (FGR) between 32 and 34 weeks, passive smoking exposure and NT.

The objectives of the study were explained to women and a written consent form was obtained from all participants. All of them participated in the study voluntarily and they were free to withdraw the study at any time. The study was approved by the Regional Medical and Bioethical Research Committee of Isfahan University of Medical Sciences (grant No. 395391, ethical code No. of IR.MUI.Rec.1395.3.391).

## Results

Four thousand six hundred five pregnant women participated in this study and the incidence rate of PE was obtained 7.4%. As a result, 333 people were included in the PE group and 4209 people in the group without PE. The mean of MOM PAPP-A was equal to 1.08 ± 0.59, the mean of MOM βhCG was equal to 1.17 ± 0.79, the mean age of the mothers was equal to 28.46 ± 5.01 years old, and the mean of BMI was equal to 24.52 ± 4.07. Among these participants, 56 subjects were excluded from the study due to intrauterine death and 10 subjects declared that they are reluctant to continue their participation. Obstetric and demographic characteristics of the two groups were compared (Table 1). There was no significant difference between the two groups in terms of the mother’s and spouse’s education level, folic acid consumption before and during the pregnancy, and preceding birth interval.

**Table 1:** Comparison of maternal obstetric and demographic characteristics of the group with PE and the group without PE

Variables	Group		*P* value
	without PE *n* = 4209	With PE *n* = 333	
Average mother’s age (year)	29.89 ± 5.29	28.32 ± 4.97	0.001
Average spouse’s age (year)	33.62 ± 5.57	32.56 ± 4.89	0.001
Average mother’s BMI (kg/m^2^)	26.14 ± 5.19	24.38 ± 3.94	0.001
Average number of pregnancies	2.03 ± 1.08	1.83 ± 0.90	0.001
Average number of deliveries	0.67 ± 0.82	0.62 ± 0.74	0.17
Mean gestational age at abortion (week) in previous pregnancies	9.03 ± 4.02	9.01 ± 3.55	0.96
NT (cm)	1.43 ± 0.37	1.39 ± 0.37	0.03
Gestational age during NT ultrasound (day)	87.18 ± 3.91	86.71 ± 3.83	0.03
Weight gain during pregnancy (kg)	12.47 ± 3.56	12.57 ± 3.61	0.6
Abortion history	89 (26.7%)	760 (18%)	0.001
Intrauterine death history	7(%0.2)	30(%0.7)	0.01
History of Type 1 diabetes mellitus	12 (3.6%)	58 (1.3%)	0.004
Gestational diabetes	39(%11.7)	329(%7.8)	0.01
Passive smoking exposure	14 (4.2%)	43 (1%)	0.01
Chronic hypertension	4 (1.2%)	14 (3%)	0.03

A significant difference was observed (*P* = 0.006) in the mean of MOM PAPP-A between the two groups so that the mean of MOM PAPP-A in the PE group was equal to 1 and for the group without PE was equal to 1.09. It means that the lower level of PAPP-A was related to the chance of developing PE. Moreover, with *P* = 0.001, the mean of MOM βhCG in the PE group was equal to 1.51 and for the group without PE, it was equal to 1.14, indicating that higher levels of βhCG were related to the chance of developing PE (Table 2).

**Table 2:** Comparison of mean MOM PAPPA and MOM βhCG in the group with PE and the group without PE

Variables	Group		*P* value
	Without PE	With PE	
MOM PAPPA Mean (SD)	1 (0.658)	1.09 (0.585)	0.006
MOM βhCG Mean (SD)	1.51 (1.15)	1.14 (0.745)	0.001

The relative risk of PE was calculated for MOM PAPP-A <  0.4: RR = 2.49, with 95% of Confidence interval (CI) (1.86–3.36), *p* = 0.001, and RR = 4.02 for MOM βhCG > 3, with 95% of CI (3.03–5.35), *p* = 0.001(Table 3).

**Table 3:** The RR and OR of (PE) based on PAPP-A and βhCG values

Variables	RR and OR with 95% of Confidence Interval		*P* value
MOM PAPP-A < 0.4	Crude RR	2.49 (1.86–3.36)	< 0.001
	Crude OR	2.80 (1.97–3.98)	< 0.001
MOM βhCG > 3	Crude RR	4.02 (3.03–5.35)	< 0.001
	Crude OR	5.13 (3.52–7.48)	< 0.001

Then, the effect of confounding variables was controlled by logistic regression. The adjusted odds ratio of PE was calculated for MOM PAPP-A <  0.4, and it was obtained as OR = 2.09, with 95% of CI (1.4–3.11), *P* = 0.001, and also it was obtained as OR = 5.65 for MOM βhCG > 3, with 95% of CI (3.74–8.53), *p* = 0.001(Table 4).

**Table 4:** The PE odds ratio after confounding variables adjusted with PAPP-A and βhCG values

Variables	OR (95% of CI)	*P*-value
Pregnant woman’s age (years)	0.92 (0.88–0.96)	0.001
Spouse’s age (years)	1.05 (1.01–1.10)	0.01
Mother’s BMI	0.91 (0.89–0.94)	0.001
Abortion history	1.38 (1.01–1.83)	0.23
FGR at 32–34 week	3.68 (2.38–5.69)	0.001
Passive smoking exposure	2 (0.99–4.05)	0.53
NT (cm)	0.69 (0.52–0.92)	0.01
MOM PAPP-A < 0.4	2.09 (1.4–3.11)	0.001
MOM βhCG > 3	5.65 (3.74–8.53)	0.001

## Discussion

This cohort study was conducted on 4605 pregnant women to evaluate PAPP-A and βhCG levels as the predictive factors of PE in the first trimester among Iranian women. The findings of the present study revealed that low Levels of PAPP-A and high levels of βhCG were significantly associated with an increased risk of PE.

Similar to the present study, Gomes et al. (2017) reported a significant statistical relationship between PAPP-A < 10th percentile and the chance of developing PE with 0R of 4.13 (1.15–14.80) and *p* = 0.03, a sensitivity of 3.33, a specificity of 89.1, a positive predictive value of 3.9 as well as the negative predictive value of 99.0 [12]. Patients who developed PE had significantly lower PAPP-A (0.79 vs. 1.19, *P* <  0.001) [9]. The median of MOM PAPP-A in women with PE was lower than the control group 0.79 vs. 0.99 and *P* < 0.01 [13]. The results of a case-control study showed that PAPP-A level was moderately associated with the development of PE the Area Under the Curve (AUC) 0.57; 95% of CI (0.53–0.61) and PAPP-A < 0.4 MOM was observed in 7.7% of the participants and it was found to have poor predictive values for PE Detection Rates 9.8%; Positive Predictive Values 6.3% [14].

Inconsistent with the present study, Ranganathan found no significant correlation between MOM PAPP-A < 0.4 and the chance of developing PE (*p* = 0.6) [7]. Furthermore, Mikat et al. stated that there is no significant difference between the PAPP-A concentration in pregnancies complicated by PE compared to uncomplicated pregnancies [6]. There was no relationship between the level of the biomarkers in the first trimester and development of PE [15].

Liu et al. (2016) showed that the serum level of MOM βhCG was higher in patients with PE [16]. They also analyzed the subgroup in terms of the ethnicity and they found that the serum levels of MOM βhCG were higher in Caucasian as well as in Asian with PE. Consistent with the present study, Di Lorenzo et al. (2012) stated that in the patients with PE, the βhCG levels were higher than those with a normal pregnancy [17]. The serum βhCG level was significantly higher in subsequently developed PE pregnancies than in uncomplicated pregnancies OR = 2.12, 95% of CI (1.07–4.21 *p* = 0.03) [6]. Contrary to the present study, high levels of βhCG in the first trimester were found to have no effect on Pregnancy-Induced Hypertension *P* = 0.628, 95% of CI (0.68–1.26), RR = 0.93 [8]. The high and the low levels of βhCG are not related to adverse outcomes of pregnancy [12].

The prospective nature of this cohort study involved the consideration of the inclusion and the exclusion criteria and all the obstetric and demographic variables. Moreover, it was possible to follow-up the patients and control the confounding variables by logistic regression. The larger sample size and a random selective population of women made it possible to find a sufficient PE rate and a real statistical investigation resulting in the higher precision of the study.

Since the number of samples was very high, researchers unable to pay for screening tests due to the lack of financial compliance, and this was considered as a limitation of this study. Thus study was conducted on pregnant women who performed the screening tests in different laboratory by themselves and were volunteers to participate in the study. Although, the screening of the fetal anomalies of the volunteers was carried out in different laboratories, but it should be mentioned that all the laboratories in Iran were required to standardize the MOM based on the software approved by the Ministry of Health and Medical Education (BENETECH, Canada and ALPHA, England). Furthermore, all the laboratories used the DEMEDITEC kits and cobas e 411 kits for performing the ELISA methods and the electro quantitative luminescent test, respectively.

## Conclusion

The results of this study showed that low levels of PAPP-A and high levels of βhCG are the predictors of PE, so that MOM PAPP-A < 0.4 causes the 2.09 times increase in the chance of developing PE and MOM βhCG > 3 causes the 5.65 times increase in the chance of developing PE. Therefore, the high levels of βhCG and low levels of PAPP-A were found to be the warning signs which can be considered as a screening method for early diagnosis of PE.

## Data Availability

The datasets used and analyzed during the current study are available from the corresponding author on reasonable request.

## Abbreviations

AUC: Area Under the Curve
BMI: Body Mass Index
CI: Confidence Interval
CRL: Crown–Rump Length
FGR: The Fetal Growth Restriction
Free βhCG: Freeβ-Human-Derived Chorionic Gonadotrophic hormone
h: hour
HELLP: H (hemolysis) EL (elevated liver enzymes**)** LP (low platelet count)
MOM: The Multiples of the Median
NT: Nuchal Translucency
OR: Odds Ratio
PAPP-A: Pregnancy-Associated Plasma Protein A
PE: Preeclampsia
RR: Relative Risk
